# Croton Chloral Hydrate

**Published:** 1874-08

**Authors:** J. Barney Yeo

**Affiliations:** M.R.C.P.


*THE LONDON LANCET—April,* 1874:

Croton Chloral Hydrate.—*By J. Barney Yeo, M.B., M.R.C.P.* * * * I venture to think my clinical observations will, so far as they have gone, support the following conclusions:

1. In croton chloral hydrate we possess a remedy of remarkable efficacy in some cases of neuralgia of the branches of the nervous trigeminus. I do not remember ever seeing a case similar to case 6, class 1, so completely relieved by any medicine, without producing the least drowsiness.

2. That it also has the power of affording relief in other obstinate forms of neuralgia.

3. That it is of use in certain cases of diffused muscular pain. I would call attention especially to the case of muscular pain after gonorrhœa. But more evidence is needed to confirm this observation.

4. That it has but little appreciable effect in purely rheumatic cases.

5. That in cases of localized pain and other nervous symptoms, which we find in the class of persons we are in the habit of calling hysterical, this drug is of little or no use. I would call attention, however, to the relief it afforded in a case of dysmenorrhœa, and suggest that those who are in the habit of seeing many such cases should give this drug a trial.

6. Its efficacy in procuring sleep seems very variable in moderate doses. Two grains will produce sleep in some sensitive females, and ten grains will fail to produce even drowsiness in unsensitive males.

7. Of its value in relieving some forms of irritative and spasmodic cough I have had abundant evidence. If I may trust to the short experience I have hitherto had of it, I should say there is scarcely any remedy that is likely to prove more valuable for the relief of the distressing night cough of chronic phthisis.

Finally, as to the dose. I am satisfied that in dealing with this substance we must give an unusually wide range to the dose, for its effects vary greatly, not only on the human subject, but also, as I shall show hereafter, on the lower animals. The doses I have given varied from one to ten grains. In delicate females I have found very decided effects from doses of two and three grains. In strong males a dose of ten grains is often required to produce any appreciable effect. As might have been expected, persons who have been accustomed to the use of anodyne medicine require larger doses than others. The dose must also be proportionate to the severity and long-continuance of the pain. I would advise that it should be always given in moderate and quickly repeated doses, until the amount of tolerance of the drug in each particular case has been discovered. In severe neuralgias, from two to five grains may be given every hour, or the smaller dose every half-hour, until fifteen grains have been taken. At present I do not think it safe to go beyond this dose.

